# Global epidemiological trends in the incidence and mortality for melanoma

**DOI:** 10.1093/skinhd/vzae013

**Published:** 2025-01-20

**Authors:** Nicola Cirillo

**Affiliations:** Faculty of Medicine, Dentistry, and Health Sciences, The University of Melbourne, Parkville, VIC, Australia

## Abstract

There is concern that increased surveillance is leading to the overdiagnosis of indolent melanomas that are not destined to be lethal. As overdiagnosis can only be appreciated at a population level, we analysed current and historical population trends of melanoma incidence and mortality worldwide. Epidemiological trends from GLOBOCAN data show signatures typical of overdiagnosis, with the magnitude of increased diagnoses far outpacing mortality for melanoma in most countries.

Dear Editor, In the last few decades, technological advances have enabled physicians to advance the time of diagnosis and detect cancer early, before it produces signs and symptoms.^1^ It is not surprising, therefore, that people who undergo skin screening subsequently experience higher rates of biopsies; in turn, increased scrutiny leads to more diagnoses of cutaneous melanoma.^2^ This would not be an undesirable outcome if it determined a reduction in mortality (or late-stage/metastatic disease as a proxy) owing to early detection. However, there is concern that increased surveillance is leading to the overdiagnosis of indolent melanomas that are not destined to be lethal. While an earlier detection might still contribute to reduction of morbidity, a recent study from Denmark using comprehensive national data reported that the surge in melanoma incidence was not associated with an increase in mortality.^3^ Upward trends in diagnostic activity and lowered diagnostic thresholds were possible causes of this inflation;^3^ ultimately, both practices can lead to melanoma overdiagnosis—the difference between the apparent amount of melanoma (observed incidence) and the true amount of clinically relevant disease (true melanoma occurrence). It is estimated that the majority of (White) patients with melanoma are overdiagnosed in the USA^4^ and Australia.^2^

The epidemiological hallmark of overdiagnosis is an increase in the incidence of disease in spite of stable mortality,^5^ where the latter is viewed as a marker for stable true cancer occurrence. As there is robust evidence that in the general population ecological studies are the most appropriate approach for quantifying and monitoring overdiagnosis,^6^ here we scrutinized available population data for melanoma.

Age-standardized incidence (ASIR) and mortality (ASMR) rates for melanoma worldwide were analysed using GLOBOCAN data.^7^ The goodness of fit (*R*^2^) for incidence and mortality data points for 2022 was assessed by best fit of the regression curve for linear (mortality increases proportionally with incidence), exponential (mortality increases exponentially with incidence) and logarithmic (mortality increases logarithmically with incidence) distributions.

Trends in incidence over time are expressed as incidence rate ratios (IRRs, age 0–84 years) between baseline (first historical data point) and current (most recent data point considered), and trends in mortality are expressed as mortality rate ratios (MRRs, age 0–84 years).

Incidence and mortality for melanoma of skin in 2022 were plotted for 162 countries and territories (Figure 1a). Differences were noted in the incidence : mortality ratios in different continents (Table S1; see Supporting Information). In Africa and Latin America, a linear distribution was a better fit for the regression curve (Figure 1a, d), suggesting that the ratio between diagnoses and deaths is relatively steady, as opposed to a logarithmic distribution for Europe and Asia (Figure 1c, e). The data points from Oceania and North America were insufficient to construct a reliable curve. When stratifying these same data points based on Human Development Index (HDI) segments, the best fit was a linear distribution for all segments except for the very high HDI group, where it was logarithmic (Figure S1; see Supporting Information). These data signify that in European and Asian countries, as well as in those with a very high HDI, additional diagnoses parallel a comparatively smaller increase in mortality at a population level.

**Figure 1:**
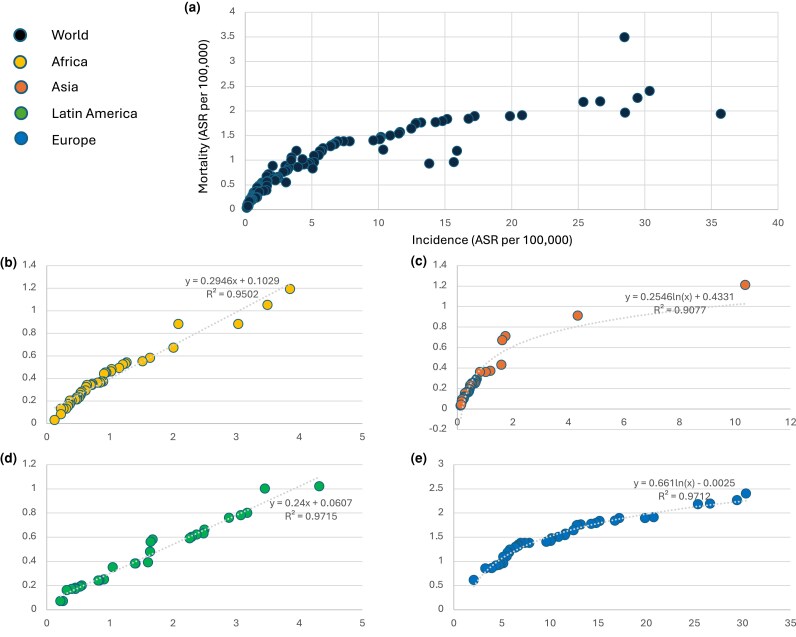
Current incidence and mortality rates for melanoma worldwide. (a) Scatter plot of incidence and mortality rates for melanoma in 2022 in 162 countries. (b–e) Correlation coefficient (*R*^2^) and regression curve with the best fit for (b) Africa, (c) Asia, (d) Latin America and (e) Europe. ASR, age-standardized rate.

To better understand the reasons underlying the observed disparities in incidence-to-mortality ratios, we examined the historical trends of incidence and mortality, where available. Of the 55 countries with some historical data points, both incidence and mortality records were available for 32 countries (Table S2; see Supporting Information). To prevent the confounding effect of improved treatment due to checkpoint-blockade immunotherapies and targeted therapies for metastatic melanoma, incidence and mortality ratios were analysed until 2014. While IRRs has increased tremendously worldwide in the last few decades, MRRs were overall stable or increased moderately. For example, in England and Wales the ASIR of melanoma (age 0–84 years) was 2.6 in women and 1.3 in men in 1971; in the following 43 years there has been a 5-fold increase in melanoma diagnoses in women and a 10-fold increase in men (IRR of 5.5 and 10.4, respectively), whereas death rates have increased <2-fold in both groups (Figure S2a; see Supporting Information). A similar trend can be observed in Scotland in the period 1975–2013 (IRR = 3.6 and 7, MRR = 1.3 and 1.8 for females and males, respectively). Scandinavian countries also show a remarkable imbalance between an increase in diagnoses and deaths from melanoma: in Sweden, there was a >7-fold increase in incidence since 1960 and a 2-fold increase in mortality (Figure S2b; see Supporting Information). Denmark witnessed a similarly upward trend in incidence in spite of a stable mortality (Figure S2c; see Supporting Information). Similar upward trends were found for New Zealand, Australia and the USA (Figure S2d, e, f; see Supporting Information), in agreement with recent reports using well-established national cancer registries.^2,4,8^ In other countries, such as Ireland, Switzerland and Japan, the imbalance between incidence and mortality appears of more modest proportion (IRR range = 1.2–2; MMR range = 0.9–2.2). Argentina, Chile, Costa Rica and Philippines were the only countries where an inverse trend was observed (MRR > IRR); however, it should be noted that melanoma is a very rare disease in these countries, and therefore the validity of these data is limited and should be interpreted with caution.

In summary, worldwide epidemiological trends demonstrate that while incidence rates for melanoma are increasing, mortality rates either increase at a remarkably slower pace or remain unchanged. Stable mortality is typically seen as an indicator of constant true cancer incidence.^3,4^ While it is conceivable that stable mortality could be the result of a simultaneous rise in true cancer incidence and advancements in treatment over time, such a perfect annual balance of these opposing factors would be an extraordinary—and unlikely—coincidence.^5^ Furthermore, even assuming that improvements in medical care were responsible for reducing melanoma mortality by a certain extent,^4^ there would still be a large divergence between diagnoses and deaths. Finally, when novel, effective treatments such as breakthroughs in immunotherapy, this generally leads to a sharp decrease in case fatalities within a short timeframe.

Thus, the global trends for melanoma suggest overdiagnosis, i.e. the detection of cancers that are unlikely to be fatal. These data are concerning because they indicate that more individuals are being diagnosed and treated for melanoma, even though the actual rate of clinically meaningful cancer occurrence has not changed. This leads to the deceptive belief that there has been a shift to more favourable metrics such as downstaging and enhanced survival, when in fact there might not be tangible improvements in health outcomes.^8^

Estimation of overdiagnosis is complex task. On an individual level, predicting overdiagnosis is virtually impossible because we cannot know how one specific lesion will behave. In fact, it is impractical or unethical to measure overdiagnosis using randomized controlled trials because once a cancer is detected it is normally treated. Overdiagnosis can therefore only be evaluated at a population level by analysing large groups of individuals through observational studies. Nevertheless, it is important to appreciate this phenomenon, given that individuals who receive an overdiagnosis may not benefit from the diagnosis and may be harmed by treatment.^4^ Recent estimates report that the degree of overdiagnosis for melanoma ranges from 29% to 60%.^9^

The observed increase in diagnoses in Australia, New Zealand, the USA, Canada and many European countries may be happening due to increased ultraviolet light exposure—but only to a limited extent.^10^ Rather, it has been proposed that it is a consequence of screening and awareness programmes for melanoma, as well as of the changing diagnostic criteria.^3,10^ Unfortunately, systematic reviews assessing the effects on morbidity and mortality of screening for malignant melanoma in the general population have failed to come up with conclusive evidence about their efficacy,^11^ suggesting that there is no solid evidence to back such screening programmes. Our study confirms that epidemiological hallmarks of melanoma overdiagnosis are present in many countries globally.

## Supplementary Material

vzae013_Supplementary_Data

## Data Availability

The data underlying this article will be shared on reasonable request to the corresponding author.

## References

[1] Welch HG, Black WC. Overdiagnosis in cancer. J Natl Cancer Inst 2010; 102:605–13. 20413742 10.1093/jnci/djq099

[2] Whiteman DC, Olsen CM, MacGregor S et al The effect of screening on melanoma incidence and biopsy rates. Br J Dermatol 2022; 187:515–22. 35531668 10.1111/bjd.21649 PMC9796145

[3] Nielsen JB, Kristiansen IS, Thapa S. Increasing melanoma incidence with unchanged mortality: more sunshine, better treatment, increased diagnostic activity, overdiagnosis or lowered diagnostic threshold? Br J Dermatol 2024; 191:365–74. 38655629 10.1093/bjd/ljae175

[4] Adamson AS, Suarez EA, Welch HG. Estimating overdiagnosis of melanoma using trends among black and white patients in the US. JAMA Dermatol 2022; 158:426–31. 35293957 10.1001/jamadermatol.2022.0139 PMC8928089

[5] Welch HG, Kramer BS, Black WC. Epidemiologic signatures in cancer. N Engl J Med 2019; 381:1378–86. 31577882 10.1056/NEJMsr1905447

[6] Carter JL, Coletti RJ, Harris RP. Quantifying and monitoring overdiagnosis in cancer screening: a systematic review of methods. BMJ 2015; 350:g7773. 25569206 10.1136/bmj.g7773 PMC4332263

[7] International Agency for Research on Cancer . Cancer over time. Available at: https://gco.iarc.who.int/en (last accessed 10 July 2024).

[8] Cirillo N . Deceptive measures of “success” in early cancer detection. Curr Oncol 2024; 31:5140–50. 39330008 10.3390/curroncol31090380 PMC11431433

[9] Bjørch MF, Gram EG, Brodersen JB. Overdiagnosis in malignant melanoma: a scoping review. BMJ Evid Based Med 2024; 29:17–28. 10.1136/bmjebm-2023-112341 37793786

[10] Welch HG, Mazer BL, Adamson AS. The rapid rise in cutaneous melanoma diagnoses. N Engl J Med 2021; 384:72–9. 33406334 10.1056/NEJMsb2019760

[11] Johansson M, Brodersen J, Gøtzsche PC, Jørgensen KJ. Screening for reducing morbidity and mortality in malignant melanoma. Cochrane Database Syst Rev 2019; 6:CD012352. 31157404 10.1002/14651858.CD012352.pub2 PMC6545529

