# Supplementary figures and images for: Global epidemiological trends in the incidence and mortality for melanoma
Source: Skin Health Dis. 2025 Jan 20;5(1):84–6. doi: 10.1093/skinhd/vzae013 (PMC11924397; doi:10.1093/skinhd/vzae013)

Mortality

very high HDI

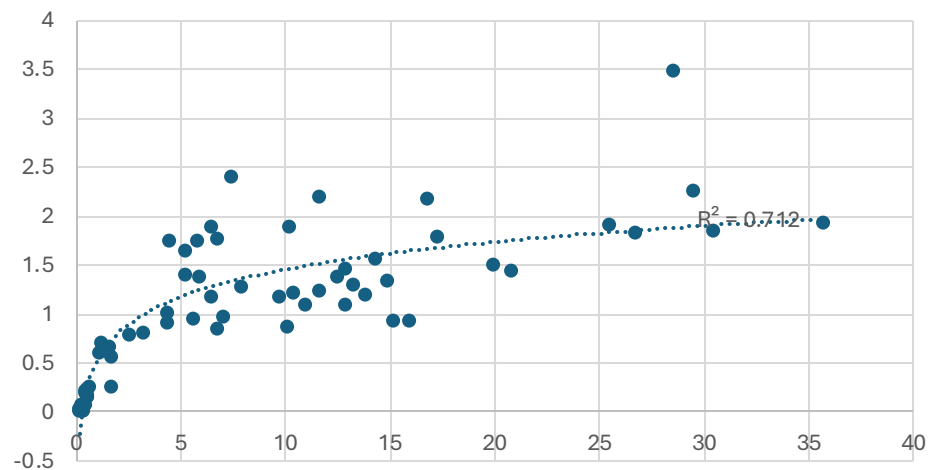

high HDI

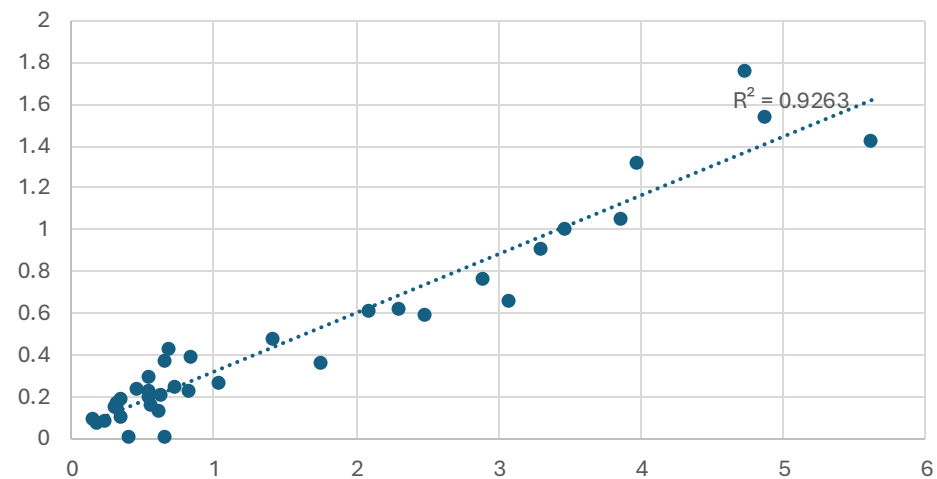

medium HDI

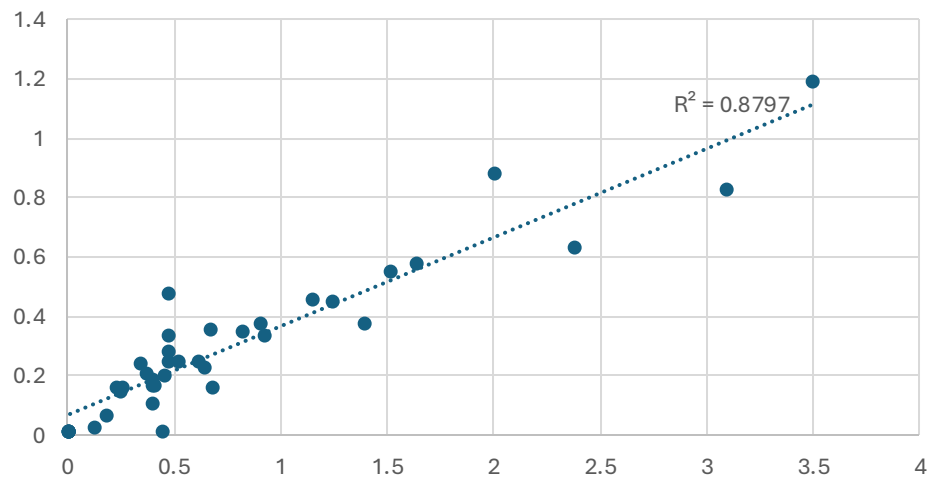

low HDI

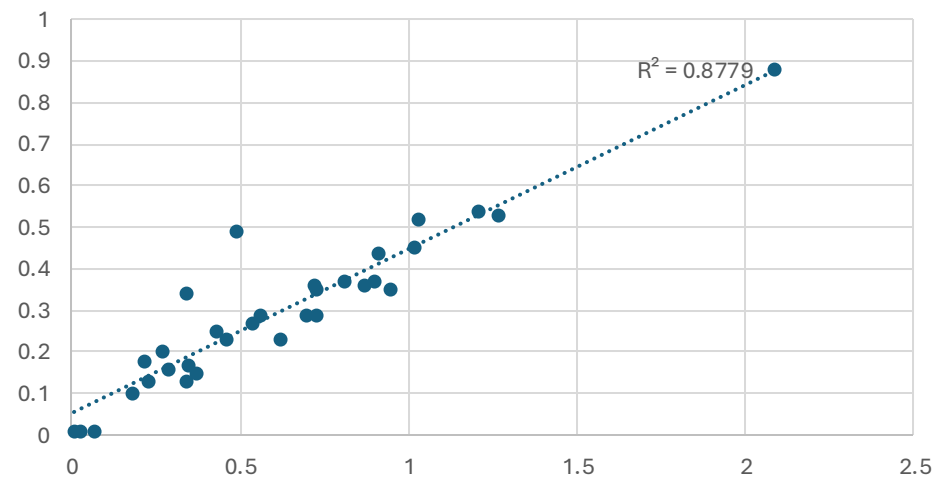

Incidence

Supplement: vzae013_Supplementary_Data [file vzae013_supplementary_data.zip › suppl Figure 1.pdf]

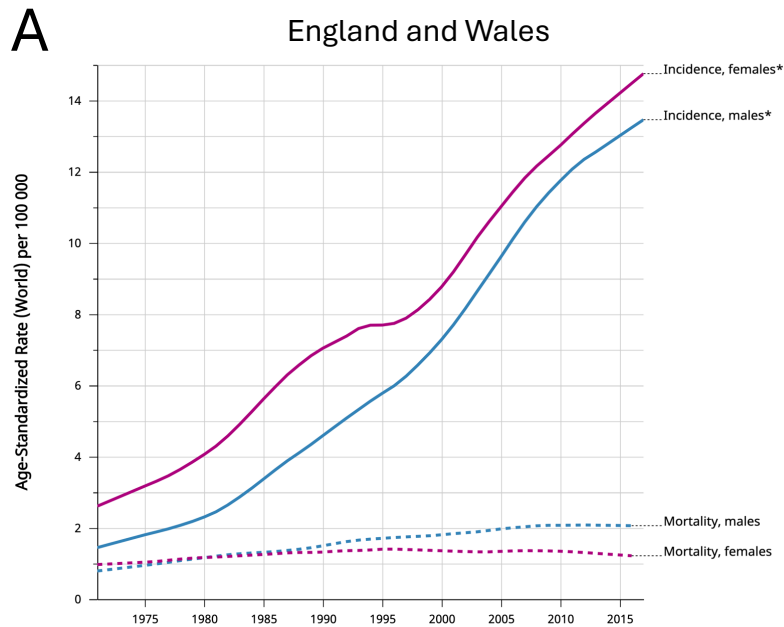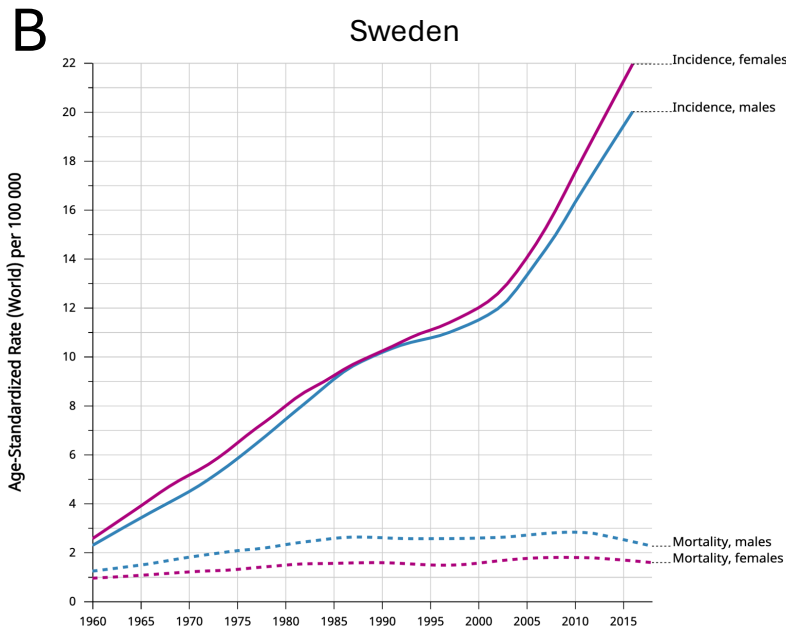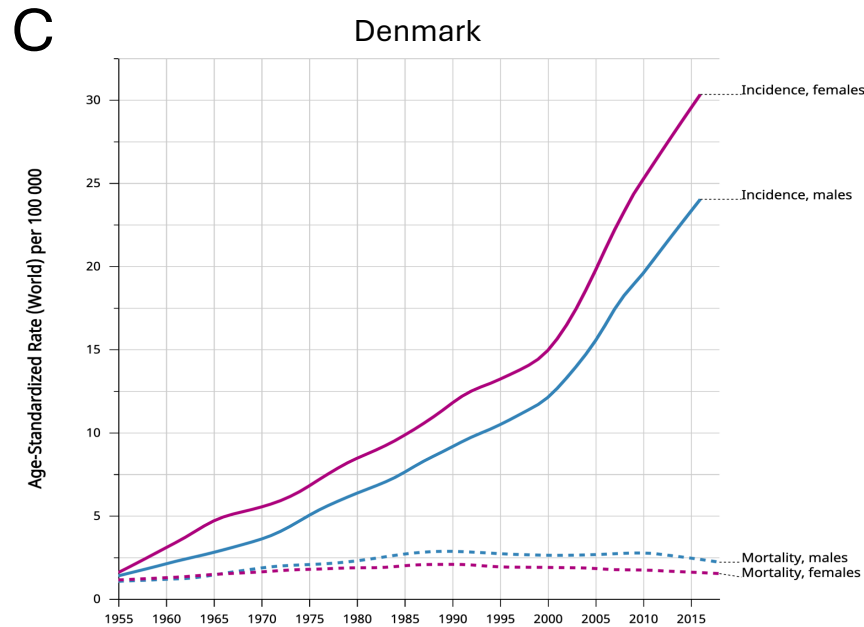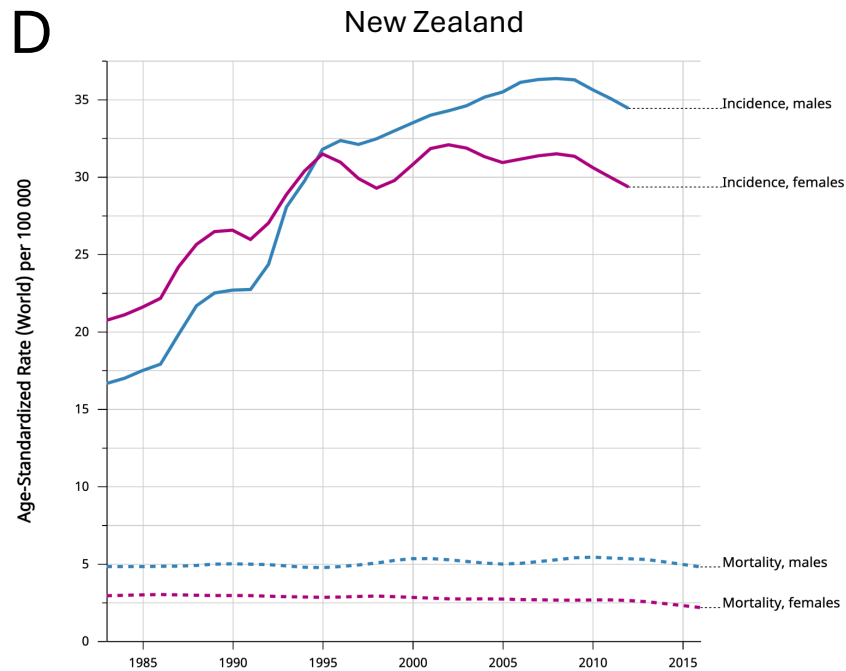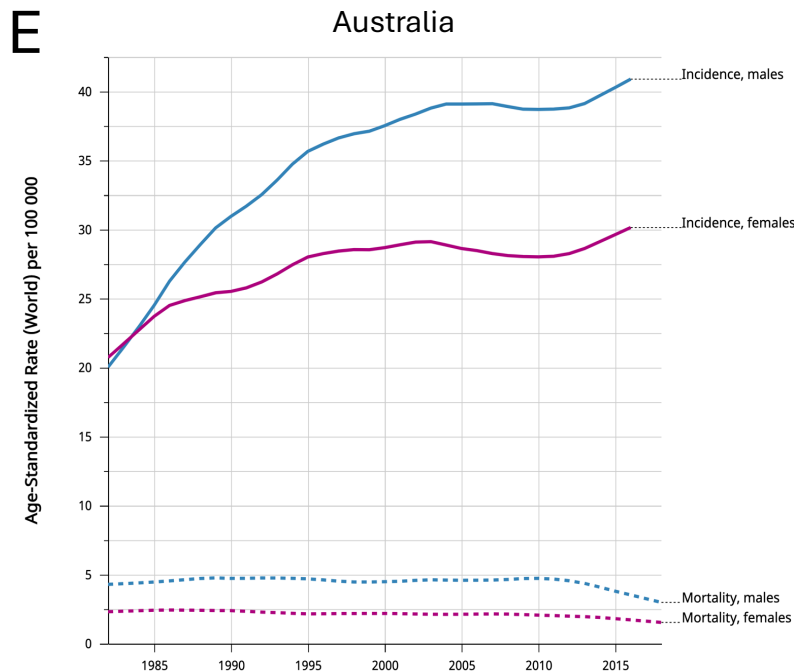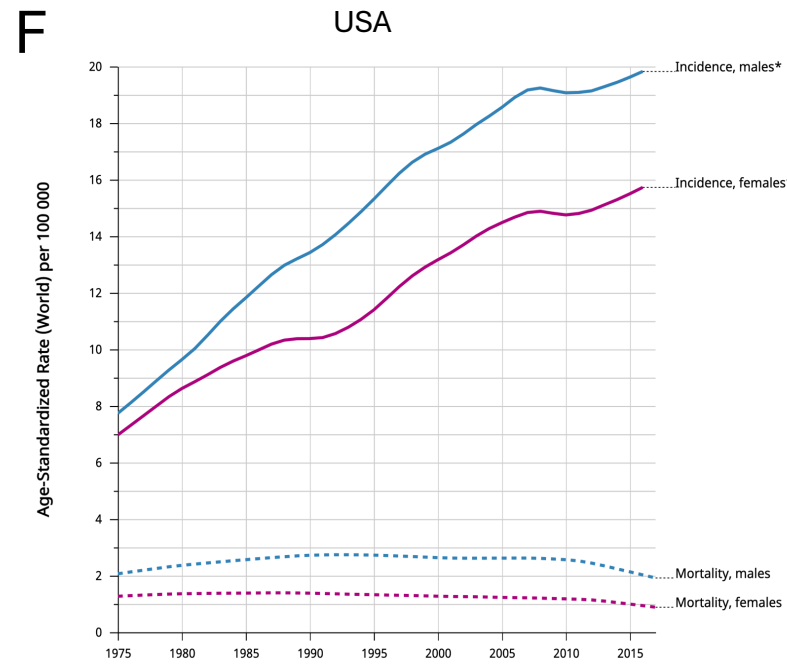

Supplement: vzae013_Supplementary_Data [file vzae013_supplementary_data.zip › Supplementary Figure 2.pdf]
